# Editorial: Is there still room for pharmacogenetics in 2023? The cases of infectious diseases and oncology

**DOI:** 10.3389/fphar.2024.1457733

**Published:** 2024-07-16

**Authors:** Alessandra Manca, Jessica Cusato

**Affiliations:** Department of Medical Sciences, University of Turin, Turin, Italy

**Keywords:** pharmacology, infectious disease, oncology, pharmacogenetics, personalized medicine

Personalized medicine has become crucial for improving patient management and clinical outcome in the Infectious Diseases and Oncology areas. In particular, the dramatic increment in resistance phenomena observed in the last 10 years, emphasizes the actual gaps and key medical needs in the fields of HIV, tuberculosis and multidrug resistant bacteria (WHO, 2024b).

Similarly, the complexity of cancer treatments, mainly related to the presence of heavy polypharmacy, with high risk of drug-drug interactions and co-morbidities in ageing patients, creates a needing for diagnostic tools, able to predict patient response to available therapies.

All these implications make the achievement of optimal drug exposure further complicated in clinical practice: most of these drugs are administered according to standard dosing regimens, which do not consider pathophysiologic, iatrogenic and, most importantly, genetic factors.

In fact, genetics is likely to affect both pharmacokinetics and pharmacodynamics of anti-infective and oncology agents in different real-life settings. Consequently, in the evolving field of tailored medicine, pharmacogenetics has become pivotal for optimizing treatment, increasing the response rate, at the same time reducing (or preventing), the development of drug-related side effects, ideally fitting into all the paradigm of medical/clinical practice, as extensively discussed in this issue of the journal.

The manuscripts discussed in this Research Topic shed light on the importance of genetic testing in managing chemotherapy dosing, analyzing the treatment outcome, and selecting patients with the higher risk of adverse effects, considering their ethnicity. Particularly, the article by Ragia et al. emphasizes the significance of implementing DPYD genotyping in Greek cancer patients receiving fluoropyrimidine-based chemotherapy. This article underlines the role of genetic variants in different populations, not considering only a global population (e.g., Caucasian): some genetic polymorphisms could have not an impact in some ethnicities, as reported for example, for abacavir and HLA-B*5701 in Sub-Saharan African population (Zhou et al., 2021). This genetic polymorphism predicts which patients treated with abacavir are more likely to develop hypersensitivity reaction, which could be severe and potentially fatal (Dean, 2012).

Maslarinou et al. recommends a comprehensive pharmacogenomic approach with dosing fluoropyrimidines, beyond the DPYD gene. Incorporating multiple genetic factors into dosage decisions, allows healthcare providers to better predict patient responses, minimizing the risks of toxicity. This polygenic algorithm has the potential to enhance safety/efficacy of fluoropyrimidine treatment for cancer patients. In fact, it is known that patients with a particular genetic background have to start fluoropyrimidines therapy with reduced dosage (50%–75%), thus with reduced plasma fluoropyrimidines exposure, which could lead to potential reduced efficacy.

Another paper in our Research Topic comes from Hurkmans et al., who investigated genetic factors associated with platinum-induced ototoxicity in childhood cancer patients. By identifying genetic markers linked to hearing loss, researchers aim to enhance the safety and efficacy of platinum-based chemotherapy through targeted interventions. Authors suggest to introduce this genetic testing, although it is not recommended by FDA (FDA, 2022), considering its potential in preventing toxicities in pediatric patients treated with platinum compounds.

Concerning infectious diseases, the article by Ulanova et al. investigates the impact of genetic variants in *NAT2*, *GSTM1*, and *CYP2E1* genes on isoniazid metabolism in tuberculosis patients. This underscores the importance of incorporating genetic factors into personalized medicine approaches for tuberculosis management, a pathology resurfaced in the last few years in our regions (WHO, 2024a). In addition, this article highlights the utility of pharmacogenetic testing in this field, although this analysis is not recommended as mandatory in the FDA table of pharmacogenetic associations (FDA, 2022).

In conclusion, in this Research Topic, recent advancements, challenges, and the potential future implications of incorporating pharmacogenetics into the treatment of infectious diseases and cancer were described. By identifying genetic Research Topic that affect drug metabolism, healthcare professionals can tailor treatment regimens considering patients variability, reducing the likelihood of severe toxicities. However, standardized guidelines, increased awareness, and improved access to testing are essential to effectively incorporate pharmacogenetic testing into clinical practice. Understanding how these genetic polymorphisms influence drug response can lead to targeted treatment strategies that improve the clinical outcome. We are confident that this issue will help to address the open question of whether there is still room for pharmacogenetics to play a significant role in the landscape of medicine in the coming years.
